# Phylogenetic and Structural Analysis of NIN-Like Proteins With a Type I/II PB1 Domain That Regulates Oligomerization for Nitrate Response

**DOI:** 10.3389/fpls.2021.672035

**Published:** 2021-05-31

**Authors:** Kuan-Ting Hsin, Tzu-Jing Yang, Yu-Hsuan Lee, Yi-Sheng Cheng

**Affiliations:** ^1^Department of Life Science, College of Life Science, National Taiwan University, Taipei, Taiwan; ^2^Institute of Biological Chemistry, Academia Sinica, Taipei, Taiwan; ^3^Institute of Biochemical Sciences, College of Life Science, National Taiwan University, Taipei, Taiwan; ^4^Institute of Plant Biology, College of Life Science, National Taiwan University, Taipei, Taiwan; ^5^Genome and Systems Biology Degree Program, College of Life Science, National Taiwan University, Taipei, Taiwan

**Keywords:** NIN-like protein, PB1, duplication, monophyly, protein–protein interaction, land plant

## Abstract

Absorption of macronutrients such as nitrogen is a critical process for land plants. There is little information available on the correlation between the root evolution of land plants and the protein regulation of nitrogen absorption and responses. *NIN*-like protein (*NLP*) transcription factors contain a Phox and Bem1 (PB1) domain, which may regulate nitrate-response genes and seem to be involved in the adaptation to growing on land in terms of plant root development. In this report, we reveal the *NLP* phylogeny in land plants and the origin of *NLP* genes that may be involved in the nitrate-signaling pathway. Our *NLP* phylogeny showed that duplication of *NLP* genes occurred before divergence of chlorophyte and land plants. Duplicated NLP genes may lost in most chlorophyte lineages. The *NLP* genes of bryophytes were initially monophyletic, but this was followed by divergence of lycophyte *NLP* genes and then angiosperm *NLP* genes. Among those identified NLP genes, PB1, a protein–protein interaction domain was identified across our phylogeny. To understand how protein–protein interaction mediate via PB1 domain, we examined the PB1 domain of *Arabidopsis thaliana* NLP7 (*AtNLP7*) in terms of its molecular oligomerization and function as representative. Based on the structure of the PB1 domain, determined using small-angle x-ray scattering (SAXS) and site-directed mutagenesis, we found that the NLP7 PB1 protein forms oligomers and that several key residues (K867 and D909/D911/E913/D922 in the OPCA motif) play a pivotal role in the oligomerization of NLP7 proteins. The fact that these residues are all conserved across land plant lineages means that this oligomerization may have evolved after the common ancestor of extant land plants colonized the land. It would then have rapidly become established across land-plant lineages in order to mediate protein–protein interactions in the nitrate-signaling pathway.

## Introduction

The evolution of land plants involved a series of adaptations to living in diverse terrestrial environments, including the evolution of roots–a key organ that allows plants to absorb macronutrients. Among macronutrients, nitrogen plays an important role in plant growth and development, and nitrates are the most abundant inorganic form of N in soils (Marchive et al., 2013; Konishi and Yanagisawa, 2014, 2019; Liu et al., 2017). Nitrates are known to be involved in plant growth and development, and to serve as signaling molecules that are involved in the primary nitrate response (PNR) (Gowri et al., 1992; Liu et al., 2017). For example, the relative growth rate of *Lactuca sativa* seedlings was 10 times higher in a nitrate-treated control than that of seedlings in a zero-nitrate treatment (Walker et al., 2001), suggesting the important role of nitrates in plant development. The PNR triggers hundreds of genes at the transcription level in response to nitrate concentration changes in the environment (Gowri et al., 1992). Molecular evidence reveals that *NIN*-like protein (NLP) transcription factors (TFs) are key players in the PNR. For example, β-glucuronidase (GUS) expression in an *NLP6* transgenic line of *Arabidopsis* can be observed after nitrate treatment, whereas no GUS signal is observed after KCl treatment (Konishi and Yanagisawa, 2013). This suggests that *NLP* may serve as a nitrate-response TF.

Nine *NLP*s (*AtNLP1*–*AtNLP9*) have so far been identified in *Arabidopsis*, and they have 34.3–72.3% similarity in their amino-acid sequences. Because of this high level of similarity, it has been proposed that there is functional redundancy within the nine *AtNLP*s, which code for nitrate-inducible gene expression (Konishi and Yanagisawa, 2019). Recently, phylogeny analysis of *NLP*s in plants has shown that these nine *AtNLP*s can be categorized into three groups, implying some functional divergence (Schauser et al., 2005; Mu and Luo, 2019; Liu and Bisseling, 2020). Group 1 comprises *AtNLP1*–*5* and may be involved in nodule formation. This hypothesis is supported by data on the protein NODULE INCEPTION (NIN) in *Medicago truncatula* (Liu et al., 2019). Group 2 comprises *AtNLP8* and -*9*, and in *Arabidopsis*, *AtNLP8* has been found to be associated with nitrate-promoted seed germination (Yan et al., 2016). Group 3 comprises *AtNLP6* and -*7*, and *Arabidopsis thaliana* NLP7 (AtNLP7) is known to be involved in regulating the expression levels of nitrate-inducible genes (Castaings et al., 2009; Wang et al., 2009). Therefore, these three groups of *NLP*s have functionally diverged over the course of evolution, and they now play roles in either nodule formation, nitrate-signaling response, or nitrate-induced gene expression.

Based on the amino-acid sequence alignment of NLPs, three conserved regions have been identified: the nitrate-response domain (NRD), the RWP-RK domain, and the Phox and Bem1 (PB1) domain (Konishi and Yanagisawa, 2014, 2019). The NRD domain is known to mediate the activation of NLPs via a conserved serine residue (serine 205 in *AtNLP7*) when nitrate is provided (Yan et al., 2016; Liu et al., 2017). The RWP-RK motif is named for the conserved amino-acid sequence Arg-Trp-Pro-X-Arg-Lys (where X indicates any amino acid), which can bind to nitrate-responsive *cis*-elements (NREs) located in the promoter region of nitrate-inducible genes (like *NIR1*) in *Arabidopsis* (Konishi and Yanagisawa, 2010; Chardin et al., 2014; Sato et al., 2016; Mu and Luo, 2019). The PB1 domain may contain either a type I or a type II domain, or both (Mu and Luo, 2019). The type I domain comprises beta I, beta II, and alpha I, which contains a conserved lysine residue. The type II domain comprises beta III, beta IV, alpha II, and beta V. AtNLPs contain both type I and type II domains (type I/II PB1 domain), which can interact with *NLP*s that contain a type I, type II, or type I/II domain (Sumimoto et al., 2007). Interaction between two PB1 domains is mediated by four core amino-acid residues (K867, D909, D911, and E913), which may facilitate NLP–NLP homodimerization (Konishi and Yanagisawa, 2019). The homodimerization of NLPs is necessary for fully promoting nitrate-induced gene expression in the presence of nitrate (Konishi and Yanagisawa, 2019). Protein–protein interaction and quantitative polymerase chain reaction (qPCR) experiments reveal that the PB1 domain plays a key role in regulating nitrate-inducible gene expression. However, how NLP proteins interact via the PB1 domain remains unclear.

In this report, the order of divergence of *NLP* genes across land plants was reconstructed to investigate how the evolution of *NLP*s correlates with root development in land plants. The *NLP*s of bryophytes were used as the root to infer the order of *NLP* gene divergence in angiosperms. In addition, the oligomerization state and protein structure of the AtNLP7 PB1 domain were revealed by size-exclusion chromatography (SEC) and small-angle X-ray scattering (SAXS). The structure and protein–protein interaction of the PB1 domain were built and confirmed using homology modeling and site-directed mutagenesis. Combining our findings regarding the evolutionary path and the molecular structure and function of AtNLP7 in plants, we conclude that the functional divergence of NLPs may be correlated with the development of plant roots in terms of nitrate response and specialized nodule formation, which occur via similar types of molecular regulation.

## Materials and Methods

### NLP Phylogeny Reconstruction

First, the core amino-acid residues of the PB1 domain in green plants were labeled in Bioedit to identify conserved *NLP* genes (Hall, 1999). Viridiplantae is composed of green algae and land plants. In reconstructed species phylogeny, seven lineages are identified within Viridiplantae, including chlorophyte, charophytes, bryophyte, lycophyte, monilophyte, gymnosperm and angiosperm (Finet et al., 2010). We aimed to reconstruct evolutionary history of *NLP* genes across major lineages within Viridiplantae. To achieve this goal, *NLP* genes of major Viridiplantae lineages were obtained from the online Phytozome database^1^ (Goodstein et al., 2012), PLAZA gymnosperm^2^ and the database of the National Center for Biotechnology Information (NCBI). The *NLP* genes of *A. thaliana* (*AtNLP1* to *AtNLP9*) were used as templates for identifying the *NLP* genes of other species. Species selected for this purpose included *Micromonas pusilla*, *Micromonas* sp., *Marchantia polymorpha*, *Selaginella moellendorffii*, *Amborella trichopoda*, gymnosperm, basal angiosperms, monocots, and eudicots (for details see Supplementary Table 1). To conduct a comprehensive search for the *NLP* genes of the selected species, the default threshold value and tblastx were set for each search. Only sequences containing an RWP-RK domain and a PB1 domain were used for phylogeny reconstruction.

Second, all *NLP* sequences were aligned using the MUSCLE algorithm (Edgar, 2004) in MEGA v.6 (Tamura et al., 2013). Next, the resulting alignment matrix was visually refined based on amino-acid translations using Bioedit (Hall, 1999). The HKY85 + G + I model was selected as the best-fitting model based on the Bayesian information criterion (BIC) (Schwarz, 1978). Both Bayesian inference (BI) and maximum likelihood (ML) methods were used to infer the relationships between the *NLP* genes, using the PhyML 3.0 online interface (Guindon et al., 2010). The HKY85 + G + I model derived from jModelTest2 (Darriba et al., 2012) was applied. Statistical support for the nodes was assessed using an approximate likelihood ratio test (aLRT) (Anisimova and Gascuel, 2006) and a Bayesian-like transformation of the aLRT (aBayes) (Anisimova et al., 2011) for the ML and BI findings, respectively. The conventional bootstrap algorithm was conducted with 1000 replicates. To present the gene relationships clearly, we present the cladogram of the land-plant *NLP* genes in the main article, but the phylogram of the *NLP* genes is included as Supplementary Figure 1.

### *AtNLP7* PB1 Domain Construction

We first attempted to express *AtNLP7* protein. However, the full-length *AtNLP7* protein tended to form an aggregate and precipitate in our pre-test. Instead, we focused on expressing PB1 domain, which serving as protein–protein interaction domain on AtNLP7. For protein expression and purification, the wild type *AtNLP7* PB1 domain was PCR-amplified from the plasmid pDL2Nx-NLP7 PB1, kindly provided by Dr. Yi-Fang Tsay (Institute of Molecular Biology, Academia Sinica), using the PB1-*Eco*RI primer (5′-ATTCCGAATTCCCAAAGGAAGAGGCCATTGC-3′) and the PB1-*Hin*dIII primer (5′-AATTAAGCTTCTAGCAGGAGCTCCCTAGATTTGTCG-3′). It was then subcloned into the expression vector pET28a such that the recombinant protein contained a six-histidine tag and thrombin cleavage site sequences at the N-terminus. Site-directed mutagenesis was performed using the QuikChange Lightning Site-Directed Mutagenesis Kit (Agilent, Santa Clara, CA, United States) to create NLP7 PB1m1 (K867A), NLP7 PB1m2 (D909A/D911A), and NLP7 PB1m3 (K867A/D909A/D911A), using specific primers (Supplementary Table 2).

### Protein Expression and Purification

The wild type and NLP7 PB1 mutants were expressed using *Escherichia coli* strain BL21 (DE3). Cultures were incubated at 37°C to an OD_600_ of 0.4–0.6 and induced using 0.1 mM isopropyl β-D-1-thiogalactopyranoside (IPTG), then grown for 8 h at 37°C. Bacterial cells were pelleted and lysed by sonication in 100 mL of buffer A (20 mM Tris–HCl pH 8.8, 0.5 M NaCl, 10% [v/v] glycerol) for wild type NLP7 PB1 (NLP7 PB1wt) and buffer B (20 mM NaH_2_PO_4_, pH 7.4, 0.5 M NaCl) for the mutants. After sonication, the cell debris were centrifuged for 25 min at 12,500 × *g*. The supernatants were filtered through a 0.45 μm filter (Sartorius) and loaded into a 5 mL Ni^2+^-Sepharose resin column (HisTrap FF, GE Healthcare). The column was washed with 10 times the column volume of buffer A for NLP7 PB1wt or buffer B for the mutants, complemented with 40 mM imidazole. The target proteins were bound and eluted with buffer A for NLP7 PB1wt or buffer B for the mutants, complemented with 500 mM imidazole. The molecular mass of the purified 6xHis-NLP7 PB1 domain was 15.6 kDa (Supplementary Figures 2–4).

### Size-Exclusion Chromatography

For advanced purification, all proteins were passed through a Superdex S-75 column (GE Healthcare) with buffer A. The molecular mass of all four proteins was estimated in buffer A using a Superdex S-75 column and calibrated with gel filtration standard markers [Bio-Rad: γ-globulin (bovine), 158 kDa; ovalbumin (chicken), 44 kDa; myoglobin (horse), 17 kDa; and vitamin B12, 1.35 kDa]. The molecular mass of NLP7 PB1 was also determined using a Superdex S-75 column and calibrated with gel filtration standard markers in buffer C [20 mM Tris, pH 8.8, 0.5 M NaCl, 10% glycerol, 5 mM reduced glutathione (GSH), 1 mM tris(2-carboxyethyl)phosphine (TCEP)]. Buffer C was designed to reduce protein aggregation and increase the solubility of the protein samples. After SEC, all proteins were concentrated using Amicon Ultra-0.5 centrifugal filters (Merck, Darmstadt, Germany) and quantified using a DS-11 spectrophotometer (DeNovix).

### Small-Angle X-Ray Scattering

Small-angle x-ray scattering was performed at the beamline (BL23A1), National Synchrotron Radiation Research Center (NSRRC), Hsinchu, Taiwan. Protein samples were placed in a 3 mm four-loading rocking cell with Kapton and collected using three different concentrations of NLP7 PB1wt dimer: 0.9, 1.2, and 1.5 mg/mL. The order of sample loading for data collection was from lowest concentration to highest. The experimental parameters for SAXS were as follows: photon energy, 15 keV; sample thickness, 2.641 mm; distance-to-sample, 4 m. For the NLP7 PB1 domain, the scattering vector (*q*) ranged from 0.007 to 0.35 Å^–1^, where *q* = 4πsinθ/λ. Data collection was performed after a brief delay of 5 s, for the transmission to normalize, for a continuous period of 200 s of x-ray exposure.

The SAXS data were analyzed using PRIMUS (Konarev et al., 2003) to estimate the state of each protein sample in the buffer condition, and model building and three-dimensional (3D) surface reconstruction was conducted using GNOM (Svergun, 1992), DAMMIF (Franke and Svergun, 2009), and Situs 2.7.3 (Wriggers and Chacón, 2001). The homology model of the NLP7 PB1 dimer was built using Modeller 9.15 (Eswar et al., 2006), based on the template of the PKC-p62 complex [Protein Data Bank (PDB) ID: 4MJS], and fitted into a volume map using UCSF Chimera (Pettersen et al., 2004).

### Dynamic Light Scattering

The protein samples were subjected to dynamic light scattering (DLS) after advanced purification by SEC. All the mutants were stored in buffer A. However, NLP7 PB1wt was analyzed in both buffer A and buffer C. Data were collected using a Zetasizer Nano ZS DLS instrument (Malvern Instruments, Malvern, United Kingdom) equipped with 50 mW laser fiber. An appropriate refractive index, viscosity (10% glycerol), and temperature (25°C) was set for each sample.

## Results

### Divergence Order of *NLP* Genes Is in Accordance With the Evolutionary Trend of Land Plants

**FIGURE 1:**
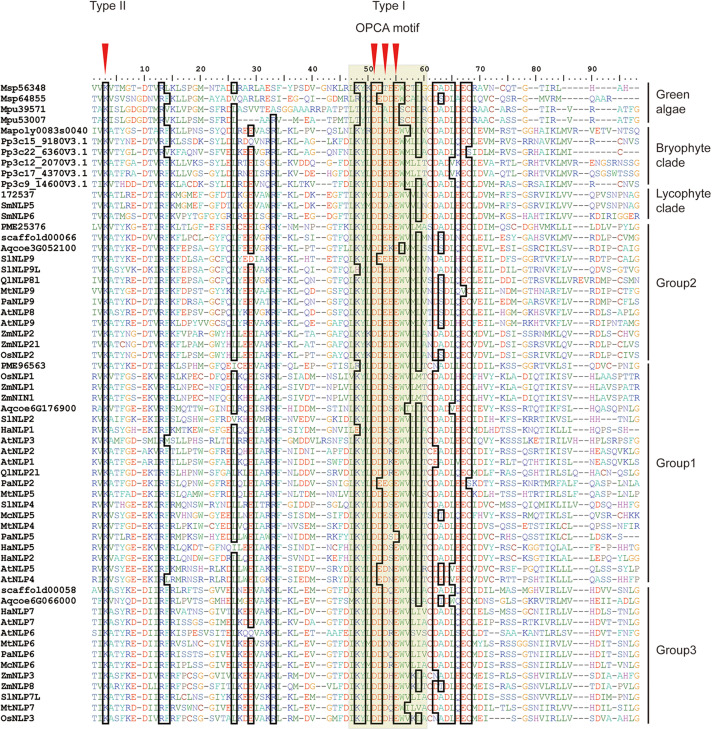
Alignment of land plant PB1 domains. First red arrow indicates core amino-acid residues in the type II motif; Second, third, and fourth arrows indicate core amino-acid residues in the type I motif. The gray hollow box indicates the OPCA motif. Sequence codes are identical to those in Figure 2.

To obtain an overview of *NLP* evolution history across Viridiplantae, we first accessed the available databases to retrieve NLP homologs from representative species, like *M. pusilla* and *M. polymorpha* (see Supplementary Table 1 for details). In total, 61 NLP homologs containing the RWP-RK and PB1 domains were obtained for the 17 selected species. At the N-terminal, a conserved core amino-acid residue, lysine (K), was observed across all the NLP proteins we used (Figure 1, red arrow). In addition, three conserved core residues within the OPCA motif at the C-terminal were identified (Figure 1). In *M. polymorpha*, only one *NLP* gene could be identified. In contrast, multiple *NLPs* were identified in the other non-vascular and vascular plants we selected.

**FIGURE 2:**
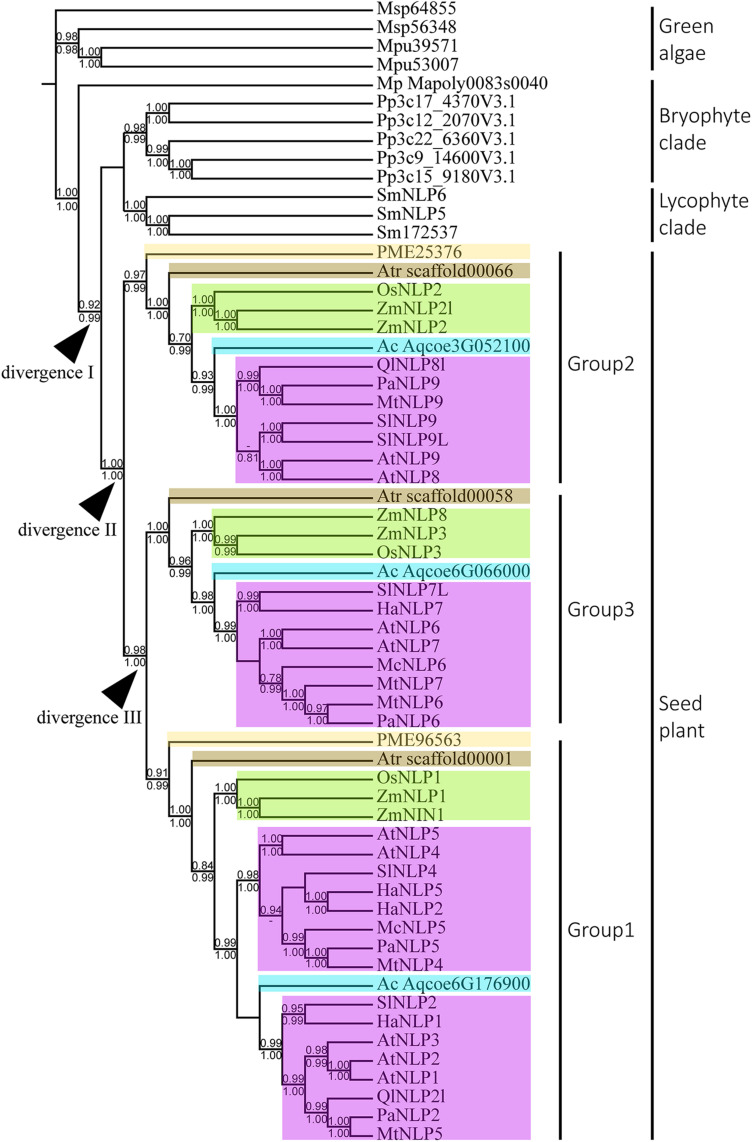
Phylogeny and divergence events of NIN-like proteins (NLPs) in land plants. The cladogram was derived from a maximum likelihood (ML) analysis and shows average branch lengths. Numbers along the branches are Bayesian inference (BI) and ML approximate likelihood values (aLRT). (–) indicates a branch-support value lower than 0.7 in either the BI or the aLRT algorithm. NLP genes of specific plant clades are colored: *Amborella trichopoda* (brown), *Oryza* and *Zea mays* (green), *Aquilegia coerulea* (blue), gymnosperm (light yellow), and angiosperms (purple). Divergences I to III indicate inferred divergence events. Mp denotes *Marchantia polymorpha*; Pp denotes *Physcomitrella patens*; Sm denotes *Selaginella moellendorffii*; Atr denotes *Amborella trichopoda*; PME denotes *Pseudotsuga menziesii*; Os denotes *Oryza sativa*; Zm denotes *Zea mays*; Ac denotes *Aquilegia coerulea*; Ql denotes *Quercus lobata*; Pa denotes *Parasponia andersonii*; Mt denotes *Medicago truncatula*; At denotes *Arabidopsis thaliana*; Sl denotes *Solanum lycopersicum*; Ha denotes *Helianthus annuus*; Mc denotes *Momordica charantia*.

To infer the divergence process of land-plant *NLP* genes, we reconstructed the evolutionary history of *NLP* genes using both BI and ML algorithms. Only nodes with BI or ML probability values over 0.8 were regarded as reliable clades. According to our criteria, three major clades can be inferred across the *NLP* phylogeny (Figure 2). The first clade appeared at the base of the phylogeny and comprised bryophytes (Figure 2, BI: –, aLRT: 0.91). Within the first clade, the *NLP* homolog found in *M. polymorpha* (Mapoly0083s0040) represented the basal lineage, following the divergence of the *NLP* genes of *Physcomitrella patens* and *S. moellendorffii*. In the bryophyte clade, the *NLP* homologs in *P. patens* and *S. moellendorffii* each formed monophyletic groups. The second clade comprised lycophyte *NLP* genes. The third clade comprised the *NLP* gene homologs found in gymnosperm and angiosperms. The divergence of the gymnosperm and angiosperm *NLP* gene homologs may have occurred in the common ancestor of seed plant, since gymnosperm, basal angiosperms, monocots, and most dicots have *NLP* genes representing all three *NLP* groups (Figure 2, divergence II, BI: 1, aLRT: 1). Within the seed plant clade, three distinct clades were identified: Group 1, Group 2, and Group 3. The Group 2 *NLP* genes diverged first (Figure 2, BI: 1, aLRT: 1), followed by the divergence between Group 3 and Group 1 *NLP* genes (Figure 2, divergence III, BI: 0.98, aLRT: 1). In Group 2, the *NLP* homolog that diverged first was that of *Pseudotsuga menziesii* PME25376, which was followed by the divergence of the *NLP* homolog in basal angiosperm, monocots, then the divergence of that in the basal dicot *Aquilegia coerulea* Aqcoe3G052100, and finally the divergence of the *NLP* homologs in the other dicots. This order of divergence (basal angiosperms, then monocots, then the basal dicot, and finally the other dicots), was also seen in Groups 1 and 3 (NLP gene of gymnosperm may lost in Group 3). Our phylogeny shows that the order of divergence of the *NLP* genes is in accordance with the species divergence process that has occurred since plants colonized the land.

### The AtNLP7 PB1 Domain Exhibits a Concentration-Dependent Oligomerization State *in vitro*

**FIGURE 3:**
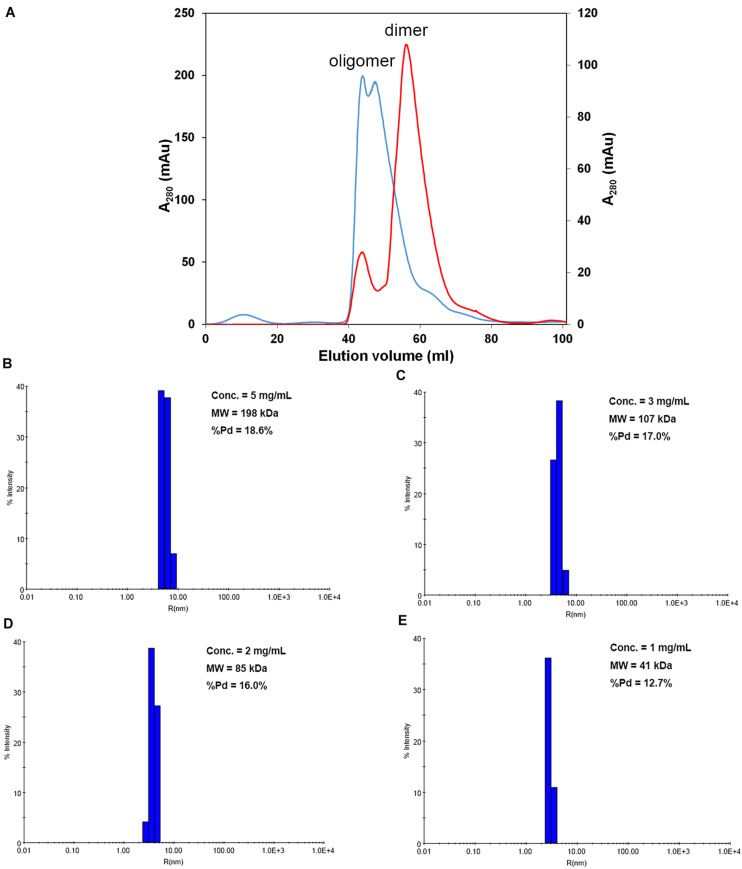
AtNLP7 PB1 tends to form a homo-oligomer *in vitro*. **(A)** Size-exclusion chromatography analysis reveals that the NLP7 PB1 domain could be eluted as a homo-oligomer in buffer A (20 mM Tris–HCl, pH 8.8, 0.5 M NaCl, 10% glycerol) or a homodimer in buffer C (20 mM Tris–HCl, pH 8.8, 0.5 M NaCl, 10% glycerol, 1 mM TCEP). The eluted profiles are colored red for the homodimer and blue for the homo-oligomer. **(B–E)** Dynamic light scattering results showed that the oligomeric state of the NLP7 PB1 domain may be correlated with its concentration. The NLP7 PB1 domain exists as a dimer in a solution with a protein concentration of 1 mg/mL, a tetramer in 2 mg/mL, and becomes an oligomer when the concentration is higher than 2 mg/mL.

We first attempted to resolve the protein structure of AtNLP7. However, the full-length AtNLP7 protein tended to form an aggregate and precipitate in our pre-test. Therefore, we tried to determine the structure of the PB1 domain, which is the protein–protein interaction domain, instead of the full protein structure of AtNLP7. During the protein purification of the AtNLP7 PB1 domain, we observed that the target PB1 protein tended to enter a dimer or oligomer state *in vitro* after SEC (Figure 3A). To examine whether the concentration of the protein solution affected the oligomerization state of the target protein, we diluted the AtNLP7 PB1 protein solution to 5, 3, 2, or 1 mg/mL. The DLS results showed that as the protein concentration increased, the molecular mass of the AtNLP7 PB1 protein increased: it was 198, 107, 85, and 41 kDa for the four concentrations, respectively (Figures 3B–E). Our SEC results thus show that the oligomerization state of AtNLP7 PB1 is affected by the concentration of its solution.

### Small-Angle X-Ray Scattering Confirmed That the Lowest Concentration of the NLP7PB1 Domain Formed a Homodimer

**FIGURE 4:**
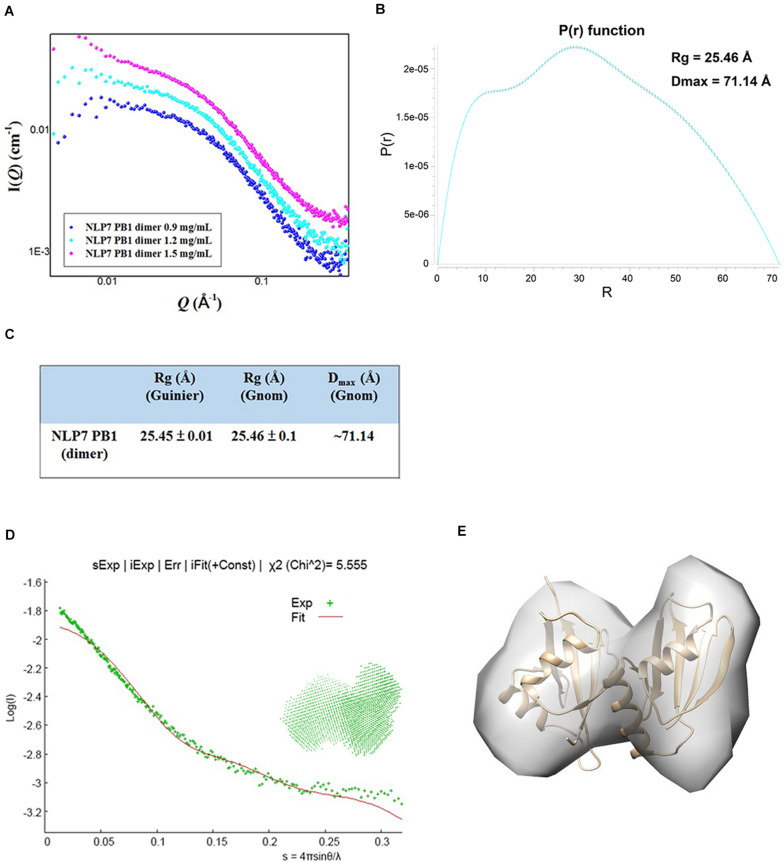
Molecular shape of NLP7 PB1, as determined using small-angle X-ray scattering (SAXS). **(A)** SAXS curves for the NLP7 PB1 domain at different protein concentrations: 0.9, 1.2, and 1.5 mg/mL. The signal for the 1.5 mg/mL solution suggests protein aggregation. **(B–C)** The radius of gyration (R_*g*_) and maximum particle size (D_*max*_) for the NLP7 PB1 domain were estimated based on a Guinier approximation in PRIMUS and the distance distribution function, P(r), in GNOM. The P(r) function curve suggests that the NLP7 PB1 domain may adopt a dumbbell-like shape. The NLP7 PB1 domain has an average R_*g*_ of 25.45 Å and a D_*max*_ of 71.14 Å. **(D)** Scattering curve (green points, experimental data) for the NLP7 PB1 domain with scattering vector values (*q*) of 0.007–0.35 Å, where *q* = 4πsinθ/λ. The experimental data were fitted with a curve (red) based on the low-resolution dummy atom model. **(E)** Superimposition of the three-dimensional structure envelope (gray) reconstructed from the dummy atom model in Situs 2.7.3 and the homology model (gold) built in Modeler 9.15 (template: 4MJS). This shows that the NLP7 PB1 domain exists in a dimeric form in solution.

The SAXS technique has been widely used to monitor molecular size, shape, and aggregation state (Kikhney and Svergun, 2015). Our previous results had suggested that the AtNLP7 PB1 domain protein formed a homodimer at the lowest protein solution concentration (1 mg/mL). To obtain a comprehensive understanding of the AtNLP7 PB1 domain protein, the protein solution was diluted to 0.9, 1.2, and 1.5 mg/mL for SAXS data collection (Figure 4A). The SAXS data processing in Primus showed that the NLP7 PB1 domain tended to aggregate when its protein concentration reached 1.5 mg/mL. These results were consistent with those from the DLS analysis (Figure 1B–E). Furthermore, the distance distribution function for NLP7 PB1 (the P(r) plot in GNOM) adopted a dumbbell-like shape (Figure 4B). The results could be used to estimate an average radius of gyration (R_*g*_) of 25.45 Å and a maximum particle size (D_*max*_) of 71.14 Å (Figure 4C) for the protein. Based on the DAMMIF calculation, a dummy atom model was generated using *ab initio* modeling (Figure 4D) and a low-resolution 3D structural envelope was reconstructed using Situs 2.7.3. The structural envelope of NLP7 PB1 had a dumbbell shape. We also built a structural model for NLP7 PB1 by homology modeling using the template of the PKC-p62 complex (PDB ID: 4MJS), a PB1–PB1 domain interaction. A dimeric form of NLP7 PB1 was generated by homology modeling and superimposed onto the 3D structural envelope from the SAXS analysis using Chimera. The superimposition result indicated that NLP7 PB1 should exist as a homodimer *in vitro* (Figure 4E).

### The AtNLP7 PB1 Domain Dimer Is Formed by Salt-Bridge Interactions

**FIGURE 5:**
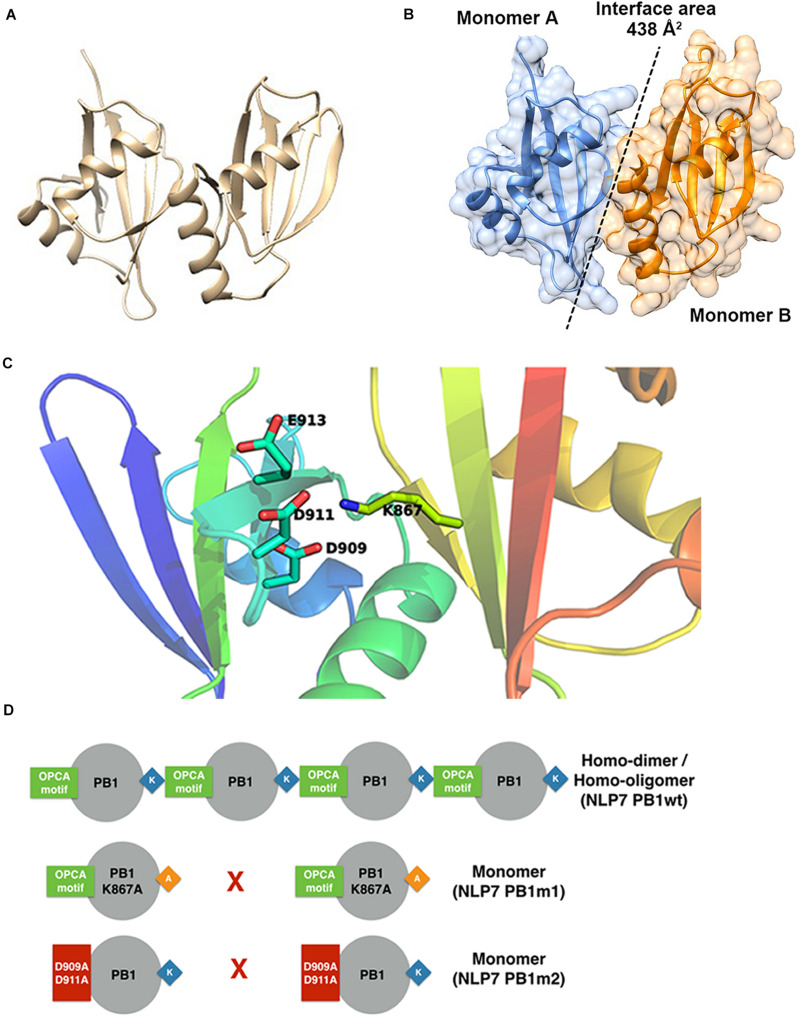
Structural model for NLP7 PB1 indicates the residues in the dimeric interface. **(A)** A structural model of the dimeric NLP7 PB1 domain was built using homology modeling with a structure template (PDB ID: 4MJS). **(B)** The interface area of the NLP7 PB1 dimer was approximately 438 Å^2^, calculated using the DALI server. Monomer A is colored blue and monomer B is colored orange. **(C)** From the structural model, the residues K867 (type I motif) and D909/D911/E913/D922 (type II motif) of NLP7 PB1 are involved in salt-bridge interactions to form the homodimer. **(D)** The proposed model shows that NLP7 PB1 could form homodimers or homo-oligomers via the interactions between the K867 and OPCA motifs. Two mutant lines, NLP7 PB1m1 (with K867A) and NLP7 PB1m2 with (D909A and D911A), were used to confirm the important roles of these motifs in the interaction between two PB1 domains.

Based on the model of the AtNLP7 PB1 dimer, we used the PDBePISA server (Krissinel and Henrick, 2007) to analyze the interface and reveal possible interactions (Figure 5A). The results showed that the interface area of the NLP7 PB1 dimer model was approximately 438 Å^2^ (Figure 5B) and that the dimerization of NLP7 PB1 occurs via salt-bridge interactions between K867 (a positively charged residue) and the OPCA motif (negatively charged residues: D909/D911/E913/D922) (Figure 5C). In addition, there are several extra hydrogen bonds and salt-bridge interactions between Phe877 and Ser881 in the positively charged interface and Glu925 and Leu916 in the negatively charged interface. These may contribute to the dimerization of the NLP7 PB1 domain via weak interactions.

### Interactions Between the K867 and OPCA Motifs Play Pivotal Roles in Oligomerization of the NLP7 PB1 Domain

**FIGURE 6:**
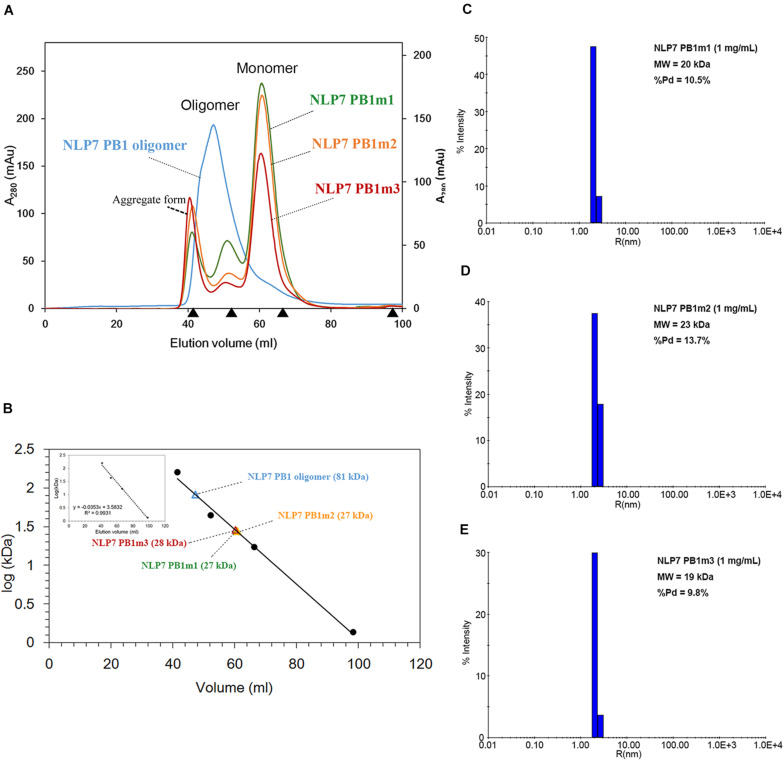
The NLP7 PB1 oligomer is maintained by the interaction between type I and II motifs. **(A)** Size-exclusion chromatography of NLP7 PB1wt (blue), PB1m1 (K867A, green), PB1m2 (D909A/D911A, orange), and PB1m3 (K867A/D909A/D911A, red) indicated that NLP7 PB1wt forms an oligomer, whereas all mutants were eluted in monomeric form in solution. **(B)** The molecular weights of NLP7 PB1wt and the mutants were estimated based on the calibration curve for protein standard markers. **(C–E)** Consistent with panels **(A,B)**, dynamic light scattering showed that NLP7 PB1m1, PB1m2, and PB1m3, in which the hypothetical interactive residues had been mutated, existed as monomers in solution.

Based on the 3D structure envelope and homology model (Figures 4E, 5A,C), we hypothesized that the dimerization of NLP7 PB1 should depend on the interaction between the K867 residue (type II motif) and OPCA (type I motif). Hence, we mutated the putative interactive residues (PB1m1, K867A; PB1m2, D909A/D911A; PB1m3, K867A/D909A/D911A) to disrupt the dimerization or oligomerization of NLP7 PB1 (Figure 6D). Indeed, the SEC results showed that all the mutants were eluted as monomers (Figures 6A,B). We thus surmised that the K867 residue plays a major interacting role in the positively charged interface to maintain the oligomerization/dimerization. Additionally, we utilized DLS to estimate the molecular weight and homogeneity of all protein samples. These were 81 kDa for NLP7 PB1wt, 27 kDa for NLP7 PB1m1, 27 kDa for NLP7 PB1m2, and 28 kDa for NLP7 PB1m3. Also, all the samples had polydispersity values lower than 20%, indicating that they formed a homogeneous (monodispersed) system after advanced purification (Figures 6C–E). Our SEC and DLS results thus provide evidence that the oligomerization/dimerization of the NLP7 PB1 domain is mediated by its two functional motifs.

## Discussion

### Presence of Conserved Residues and the OPCA Motif in the PB1 Domain Across Land Plant Lineages Suggests Ancient Origin of Protein–Protein Interaction Function of PB1 Domain in Regulating Nitrate-Signaling Pathway in Land Plants

In the PB1 domain, there are four key amino-acid residues (K in the type II motif and D, D, and E in the OPCA motif) that are responsible for mediating protein–protein interactions (Sumimoto et al., 2007; Konishi and Yanagisawa, 2019). By aligning PB1 domain residues, we found that these four residues remain almost identical at the amino-acid level across bryophyte and angiosperm species, suggesting an ancient origin of and functional constraint on these four residues (Figure 1). Site-directed mutagenesis assays have shown that mutations of these four residues can inhibit PB1–PB1 interaction in both humans (Nakamura et al., 1998; Yoshinaga et al., 2003) and *Arabidopsis* (Konishi and Yanagisawa, 2019).

With extensive sampling of 61 *NLP*-like sequences from 17 representative species across bryophytes and angiosperms, we reconstructed an across-Viridiplantae *NLP* phylogeny that suggested the inference of at least four duplication events across the sampled species during the evolutionary history of *NLP* genes. The monophyly of the *NLP* homologs obtained from bryophytes and seed plant suggests that these two clades originated in the most recent common ancestor predating the divergence of seed plant (Figure 2, divergence I). Within seed plant, the *NLP* genes formed three well-supported monophyletic groups: Group 2 (aLRT: 1; BI: –), Group 1 (aLRT: 0.99; BI: 1), and Group 3 (aLRT: 1; BI: 1). Group 2 diverged first, followed by divergence between Group 3 and Group 1 (Figure 2, divergence II and III). Within each group, a general *NLP* gene divergence pattern was identified. The *NLP* gene of *A. trichopoda* (brown box) is the basal lineage, followed by divergence of monocot *NLP* genes (*Oryza* and *Zea mays*, green box), then a basal angiosperm (*A. coerulea*, sky blue box), and lastly the *NLP* genes of other angiosperms (purple box). This diversification pattern suggests that the angiosperm *NLP* genes completed their lineage sorting and diverged prior to angiosperm diversification. We identified three distinct *NLP* clades, similar to what previous studies have found (Suzuki et al., 2013; Mu and Luo, 2019; Liu and Bisseling, 2020; Wu et al., 2020). Taking the *AtNLP* genes as an example, *AtNLP8* and *AtNLP9* belong to Group 2; *AtNLP6* and *AtNLP7* belong to Group 3, and *AtNLP1*–*5* belong to Group 1, which is consistent with previous studies (Mu and Luo, 2019; Liu and Bisseling, 2020; Wu et al., 2020). Combining these findings with functional studies of *NLP*s, we postulate that the *NLP* genes underwent functional divergence following the clade divergence. For example, *AtNLP8* is known to be a master regulator of nitrate-promoted seed germination (Yan et al., 2016), *AtNLP7* is regarded as a master regulator of nitrate-inducible genes (Konishi and Yanagisawa, 2019), and *AtNLP1* may contribute to nodulation in plants not in the nitrogen-fixation clade (NFC) (Wu et al., 2020). The topology published by Liu and Bisseling (2020) suggested an ancient and independent origin of the nodulation and nitrate-fixation functions of *NLP*s in angiosperms. Contrary to their findings, our topology suggests that the nodulation and nitrate-fixation functions may be recently evolved rather than ancestral (Figure 2 and Supplementary Figure 1).

*NIN*-like protein genes obtained from selected Viridiplantae species contains two key domains, including RWP-RK and PB1 domain. RWP-RK domain was regarded as a “plant-specific” domain (Mu and Luo, 2019), suggesting RWP-RK domain originating from common ancestor of Viridiplantae. However, RWP-RK domain is identified in oomycete, but PB1 domain absent (Yin et al., 2020). Their finding suggests RWP-RK domain may evolve since the last eukaryotic ancestor. Altogether, we can hypothesize that common ancestor of Viridiplantae gained PB1 domain, forming *NLP* gene. Following species divergence, *NLP* gene maintained in few green algae lineages (e.g., *M. pusilla*) and multiple duplication events occurred after emergence of common ancestor of land plant. The lack of fern *NLP* genes in this study is due to the absence of complete *NLP* sequences in the databases we searched. In addition, the key residues for regulating the *AtNLP7* PB1 domain interaction appeared to have been conserved in land plants (Figure 1, K867 and D909/D911/E913, red arrows; Figure 5). Therefore, we postulate that these key residues of the PB1 domain evolved prior to land-plant diversification. A possible explanation for why multiple *NLP* duplicates have been preserved across land plants could be related to the wide range of environmental conditions in land-plant habitats. *M. polymorpha* occurs in moist environments where nitrates are sufficient. However, when diverse land habitats were colonized, such as savannas and mountains, the uptake of nitrogen may have been more difficult than in moist environments. In addition, the duplicated *NLP* genes may have provided opportunities for symbiosis with bacteria to form nodules facilitating nitrogen uptake, as with the Group 1 *NLP* genes of legumes (Huang et al., 2018; Liu et al., 2019).

### AtNLP7 PB1 Domain Mediates Self-Oligomerization Through Dimerization

*Arabidopsis thaliana* NLP7 is known to be a master regulator of the nitrate-signaling pathway in *Arabidopsis* (Konishi and Yanagisawa, 2013, 2014, 2019). The PB1 domain is located at the C-terminus in AtNLP7 and has been shown to be responsible for protein–protein interactions in regulating nitrate-inducible gene expression (Konishi and Yanagisawa, 2019). For example, the expression level of *NIA1*, a nitrate reductase gene, is reduced in *AtNLP7* PB1-domain mutant lines (Konishi and Yanagisawa, 2019). In addition, shoot fresh weight in PB1-domain mutant lines is lower than that in the wild type (Konishi and Yanagisawa, 2019). Even though the PB1 domain is known to mediate protein–protein interactions, the oligomerization state of this domain remains unknown. Understanding the oligomerization state of a target protein or domain allows us to gain knowledge about how traits or organs developed and differentiated. For example, hetero- and homodimer forms of the MADS box protein have been found to be associated with subtle quantitative differences in stamen shape in maize (Abraham-Juarez et al., 2020). In this study, we used SAXS and structural modeling to confirm that the AtNLP7 PB1 domain could form homodimers via salt-bridge interactions *in vitro* (Figures 4, 5). In addition, we showed that mutation of the lysine residue (K867) in the type II motif and aspartic acid residues (D909A/D911A) in the OPCA motif would change the oligomerization state of the PB1 domain from homodimer to monomer (Figure 5). Combined with the previous analysis of expression levels of *AtNLP7* PB1 mutants conducted by Konishi and Yanagisawa (2019), we hypothesize that if the homodimer form of AtNLP7 PB1 is lost, the growth of *Arabidopsis* roots would be reduced. Our findings provide insight into how AtNLP is involved, via the PB1 domain at the protein level, in the nitrate-signaling pathway in land plants.

### AtNLP7 PB1 Domain Could Also Serve as an Interaction Domain in Mediating the Auxin-Response Regulatory Network in Plants

**FIGURE 7:**
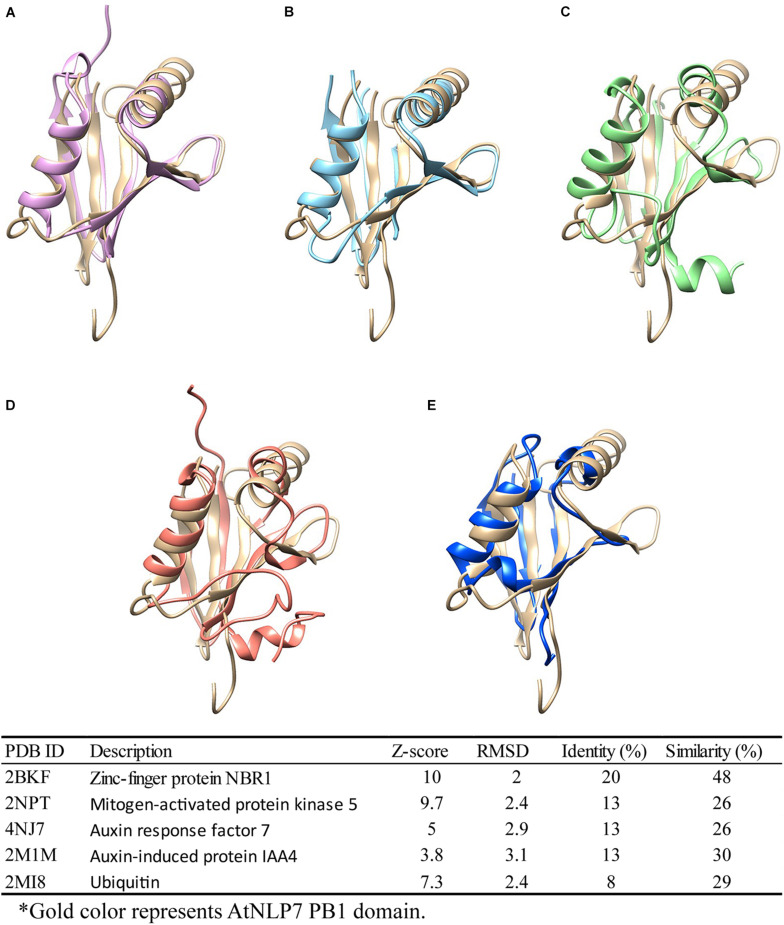
Structural comparison of NLP7 PB1 structural model with PB1-containing proteins and ubiquitin. Protein-structure searching and comparison of NLP7 PB1 domain homology using the DALI server. NLP7 (gold) is shown compared with **(A)** NBR1 (pink), **(B)** MEKK5 (light blue), **(C)** ARF7 (green), **(D)** IAA4 (orange), and **(E)** ubiquitin (dark blue), and the table summarizes the findings.

As previously reported, the PB1 domain is found in yeast, humans, and plants, and plays diverse roles in different species (Sumimoto et al., 2007). Among these species, PB1 domain-containing genes play different roles. For example, PB1 domain interaction between PB1 domain-containing p67^*phox*^ and p40^*phox*^ genes is involved in the activation of nicotinamide adenine dinucleotide phosphate (NADPH) oxidase in mammals (Cross and Segal, 2004; Sumimoto et al., 2005). In plants, PB1 domain-containing genes are involved in nitrogen fixation, nitrate signaling, and seed germination (Yan et al., 2016; Konishi and Yanagisawa, 2019). In earlier functional studies, sequence (Tian and Skolnick, 2003) or structure (Wilson et al., 2000) similarities were used to explore and assume protein functions among similar protein sequences or structures (Rost, 2002; Conant and Wolfe, 2008). Therefore, to explore the potential role of the AtNLP7 PB1 domain, we performed a structural comparison of this domain using the DALI server (Holm, 2020). We found five candidates with structural identity (Figures 7A–E), ranging from 8 to 20% (amino acid similarity ranging from 26 to 48%). Of these, Auxin response factor 7 (ARF7) and Auxin-induced protein (IAA4) attracted our attention first. Interaction between ARF7 and IAA4 was known to occur via their C-terminal domain, called Domain III/IV, which has recently been identified as a type I/II PB1 domain. This interaction facilitates their homo-/hetero-/oligomerization via the same mechanism as AtNLP7 PB1 (Korasick et al., 2014). In addition, another interesting candidate is ubiquitin, which is known to be a targeting protein that links to the lysine residue of proteins targeted for intracellular degradation (Tan et al., 2013). A recent study found that PB1 domain-containing protein kinase C was able to form hetero-oligomers to prevent biased assemblage of p62 protein (Ciuffa et al., 2015). Our structural modeling provides potential roles for the PB1 domain for further exploration of its function in plants.

## Conclusion

In summary, our phylogeny shows that divergence of angiosperm *NLP* genes occurred before angiosperm diversification. In addition, a complete lineage sorting of each group and the maintenance of duplicates within each group (except one possible loss in *A. coerulea* in Group 1) suggests that the *NLP* genes may be suitable for revealing evolutionary relationships in plants at high taxonomy levels. Furthermore, our biophysical studies and structural model of the AtNLP7 PB1 domain indicate that this domain can form either homodimers or homo-oligomers in regulating the nitrate-response network.

## Data Availability Statement

The datasets presented in this study can be found in online repositories. The names of the repository/repositories and accession number(s) can be found in the article/Supplementary Material.

## Author Contributions

K-TH, T-JY, and Y-SC: conceptualization. K-TH, T-JY, and Y-HL: data curation. K-TH and T-JY: formal analysis and writing–original draft. Y-SC: funding acquisition and supervision. K-TH, T-JY, Y-HL, and Y-SC: methodology. K-TH, Y-HL, and Y-SC: writing–review and editing. All authors contributed to the article and approved the submitted version.

## Conflict of Interest

The authors declare that the research was conducted in the absence of any commercial or financial relationships that could be construed as a potential conflict of interest.

## Footnotes

1 https://phytozome.jgi.doe.gov/pz/portal.html#

2 https://bioinformatics.psb.ugent.be/plaza/versions/gymno-plaza/

## Supplementary Material

Supplementary Figure 1 Phylogram of *NLP* genes.

Supplementary Figure 2 SDS-PAGE of purified 6xHis-tagged NLP7 PB1 domain. SDS-PAGE reveals that the molecular weight of the purified 6xHis-tagged NLP7 PB1wt domain is 15.6 kDa.

Supplementary Figure 3 SDS-PAGE of purified 6xHis-tagged NLP7 PB1m1 domain. SDS-PAGE reveals that the molecular weight of the purified 6xHis-tagged NLP7 PB1m1 domain is 15.6 kDa.

Supplementary Figure 4 SDS-PAGE of purified 6xHis-tagged NLP7 PB1m2 domain. SDS-PAGE reveals that the molecular weight of the purified 6xHis-tagged NLP7 PB1m2 domain is 15.6 kDa.

Supplementary Table 1 List of species and sequences used in phylogeny reconstruction.

Supplementary Table 2 Primers used in the construction of the *NLP7* PB1 domain and mutants.

Supplementary Table 3 Pairwise nucleotide similarity among AtNLP7 PB1 and identified PB1 domain containg homologs.

Supplementary Data 1 Alignment of NLP genes used in this study.
