# Supplementary material for: Phylogenetic and Structural Analysis of NIN-Like Proteins With a Type I/II PB1 Domain That Regulates Oligomerization for Nitrate Response
Source: Front Plant Sci. 2021 May 31;12:672035. doi: 10.3389/fpls.2021.672035 (PMC8200828; doi:10.3389/fpls.2021.672035)
Supplement: Supplementary Figure 1 — Phylogram of NLP genes. [file Data_Sheet_1.docx]

SUPPLEMENTARY MATERIAL


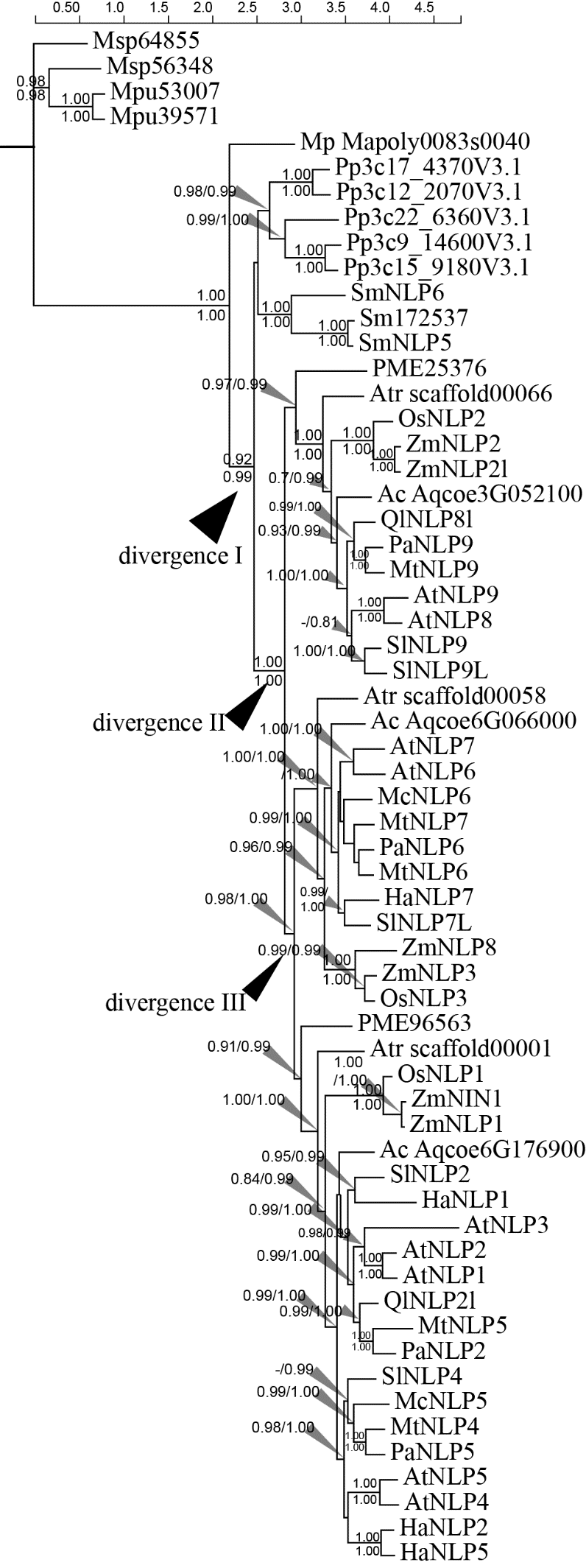


Supplementary Figure 1. Phylogram of Green plant NLP genes. Numbers along branches are BI and ML approximate likelihood value (aLRT). (-) indicates branch support value lower than 0.7 either obtaining from BI or aLRT algorithm. Sequences codes are identical to corresponding species declared in Figure 2.


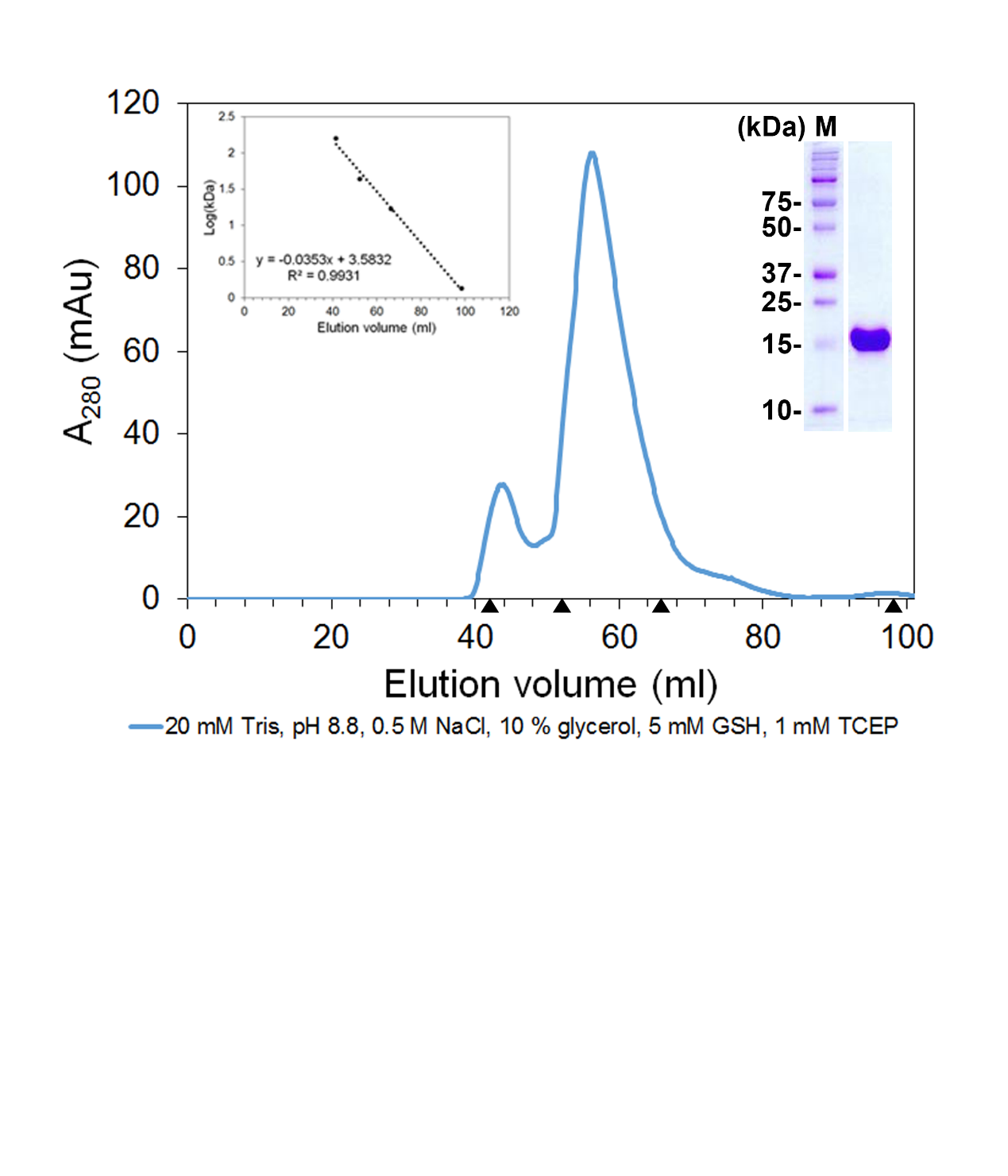


Supplementary Figure 2. SDS-PAGE of purified 6xHis-tagged NLP7 PB1 domain. SDS-PAGE reveals that the molecular weight of purified 6xHis-tagged NLP7 PB1 domain is 15.6 kDa.


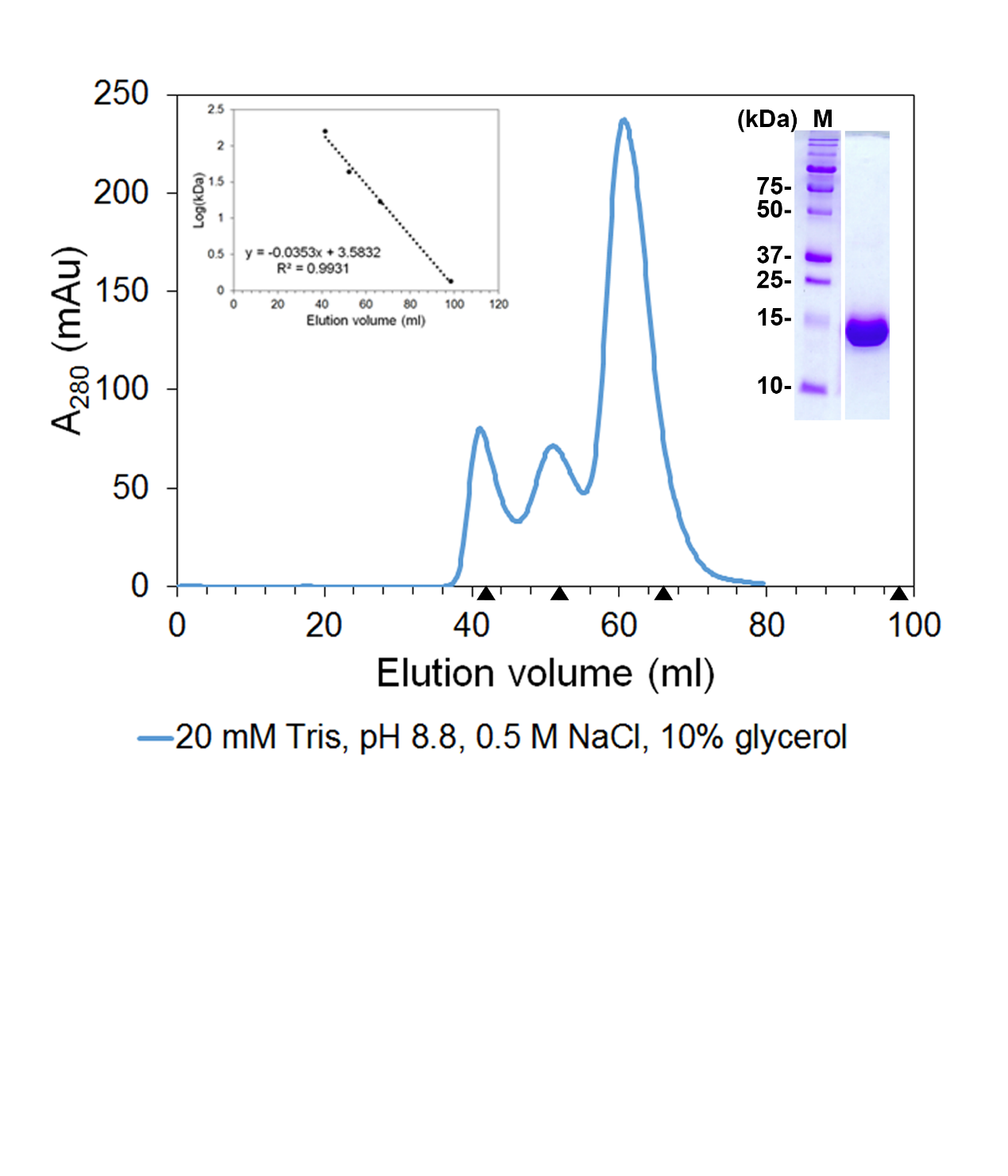


Supplementary Figure 3. SDS-PAGE of purified 6xHis-tagged NLP7 PB1m1 domain. SDS-PAGE reveals that the molecular weight of purified 6xHis-tagged NLP7 PB1m1 domain is 15.6 kDa.


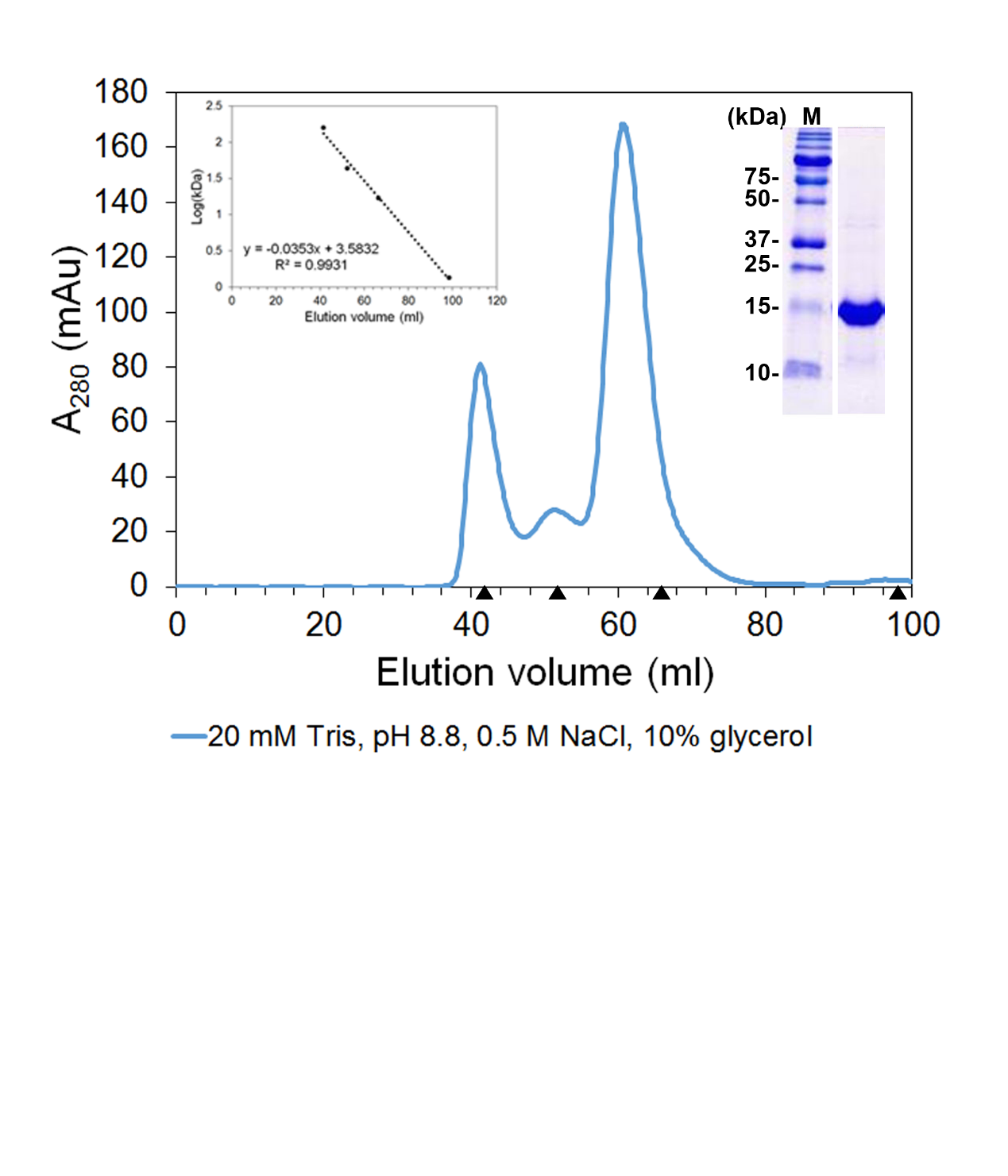


Supplementary Figure 4. SDS-PAGE of purified 6xHis-tagged NLP7 PB1m2 domain. SDS-PAGE reveals that the molecular weight of purified 6xHis-tagged NLP7 PB1m2 domain is 15.6 kDa.
